# Functional Modulation of Cardiac Form through Regionally Confined Cell Shape Changes

**DOI:** 10.1371/journal.pbio.0050053

**Published:** 2007-02-20

**Authors:** Heidi J Auman, Hope Coleman, Heather E Riley, Felix Olale, Huai-Jen Tsai, Deborah Yelon

**Affiliations:** 1 Developmental Genetics Program and Department of Cell Biology, Skirball Institute of Biomolecular Medicine, New York University School of Medicine, New York, New York, United States of America; 2 Institute of Molecular and Cell Biology, College of Life Science, National Taiwan University, Taipei, Taiwan; Duke University Medical Center, United States of America

## Abstract

Developing organs acquire a specific three-dimensional form that ensures their normal function. Cardiac function, for example, depends upon properly shaped chambers that emerge from a primitive heart tube. The cellular mechanisms that control chamber shape are not yet understood. Here, we demonstrate that chamber morphology develops via changes in cell morphology, and we determine key regulatory influences on this process. Focusing on the development of the ventricular chamber in zebrafish, we show that cardiomyocyte cell shape changes underlie the formation of characteristic chamber curvatures. In particular, cardiomyocyte elongation occurs within a confined area that forms the ventricular outer curvature. Because cardiac contractility and blood flow begin before chambers emerge, cardiac function has the potential to influence chamber curvature formation. Employing zebrafish mutants with functional deficiencies, we find that blood flow and contractility independently regulate cell shape changes in the emerging ventricle. Reduction of circulation limits the extent of cardiomyocyte elongation; in contrast, disruption of sarcomere formation releases limitations on cardiomyocyte dimensions. Thus, the acquisition of normal cardiomyocyte morphology requires a balance between extrinsic and intrinsic physical forces. Together, these data establish regionally confined cell shape change as a cellular mechanism for chamber emergence and as a link in the relationship between form and function during organ morphogenesis.

## Author Summary

As organs develop, they acquire a characteristic shape; the factors governing this complex process, however, are not understood. Shape may be sculpted by cell movement, cell division, or changes in cell size and shape, all of which can be influenced by the local environment. Here we investigate heart formation to understand how organs develop. The heart appears as a simple tube early in development; later, the tube walls bulge outward to form the cardiac chambers. Using transgenic zebrafish in which we can watch individual cardiac cells, we found that cells change size and shape, enlarging and elongating to form the bulges in the heart tube and eventually the chambers. Since the heart is beating as it develops, we asked whether cardiac function influences cell shape. Using zebrafish mutants with functional defects, we found that both blood flow and cardiac contractility influence cardiac cell shape. We propose that a balance of the cell's internal forces (through contractility) with external forces (such as blood flow) is necessary to create the cell shapes that generate chamber curvatures. Disruption of this balance may underlie the aberrations observed in some types of heart disease.

## Abstract 3

Cardiac function depends upon properly shaped heart chambers. Here the authors show that blood flow and contractility independently regulate cell shape changes in the emerging ventricle.

## Introduction

Embryonic organs acquire a characteristic size and shape during their development. Cell division and cell growth control are known to influence organ size (reviewed in [1–4]). Organ shape, on the other hand, is not simply a consequence of accumulated cell number and mass; organ shaping is also a physical process, during which tissues are pressed, pulled, and moved. Therefore, the acquisition of an organ's three-dimensional form may involve an important interplay of cell shape, cellular organization, and the physical forces that impact them [5,6].

**Figure 1:**
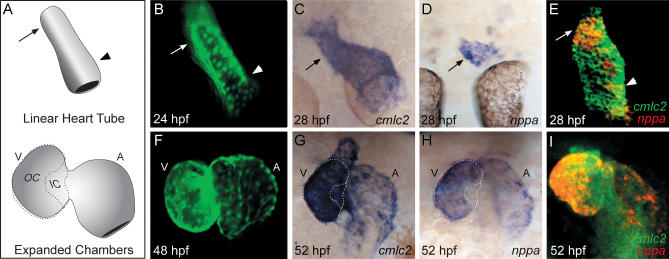
*nppa* Expression Distinguishes the OC and IC of the Zebrafish Ventricle (A) Cartoon of the zebrafish LHT (24–28 hpf) and expanded chamber (48–58 hpf) stages. The expanded ventricle (V) and atrium (A) each exhibit an OC and IC, as outlined on the ventricle. (B and F) Live images of embryos expressing *Tg(cmlc2:egfp)* in the LHT (dorsal view) and in the expanded chambers (frontal view). The arterial and venous halves of the LHT will form the ventricle and atrium, respectively [51]. Specific regions of the LHT will expand to create the OC of the ventricle ([B] arrow) and the OC of the atrium ([B] arrowhead). (C–E and G–I) Whole-mount in situ hybridization comparing expression of the myocardial gene *cardiac myosin light chain 2 (cmlc2)* with expression of *nppa* at LHT ([C–E] dorsal view) and expanded chamber ([G–I] frontal view) stages. (E and I) Fluorescent in situ hybridization depicts *cmlc2* expression in green and *nppa* expression in red. In the LHT, *nppa* expression is regionally restricted to the future OC of the ventricle ([C–E] arrow); at this stage, faint expression is also detectable in the future OC of the atrium ([E] arrowhead). In the expanded chambers, *nppa* is expressed in the OC, but absent from the IC and atrioventricular canal (G–I).

The particular shape of the embryonic vertebrate heart is critical for its normal function: the heart is composed of a series of chambers that rhythmically drive circulation, and the morphology of each chamber contributes to its functional capacity. The primitive heart begins as a simple linear tube, and this structure gradually transforms into two discrete chambers, a ventricle and an atrium (Figure 1A); in higher vertebrates, septation later divides these two chambers into four [7,8]. Transformation of the heart tube into the chambered heart requires simultaneous processes known as *looping* and *ballooning*. Looping involves bending and twisting of the heart tube in order to create asymmetry of the chambers relative to the embryonic left–right axis [7,9]. Ballooning involves the emergence of localized bulges on the tubular wall that expand to create the shapes of the cardiac chambers [8,10]. Each expanded chamber assumes a bean-like morphology, featuring two distinctly curved surfaces: a greater, convex curvature called the outer curvature (OC) and a lesser, concave curvature called the inner curvature (IC) (Figure 1A) [10–12]. In addition to their morphological differences, the OC and IC can be distinguished by the expression of curvature-specific genes (e.g., the OC gene *natriuretic peptide precursor a [nppa]*) and different physiological properties (e.g., faster conduction velocity at the OC) [10,12–14].

Formation of chamber curvatures is a critical step in the development of a functionally mature heart, yet little is understood about the mechanisms that establish chamber shape. Curvature dimensions could be the product of differential patterns of cell proliferation, cellular organization, or cell shape changes. Each of these mechanisms has been suggested to contribute to cardiac looping [15–20]. Previous studies indicate the existence of differential proliferation rates within areas of the looped chick heart [21]. However, studies in mouse argue against the notion that proliferative centers govern cardiac morphogenesis and instead favor patterns of oriented cell division as a mechanism for generating chamber form [22]. Other studies in the chick have found that apical cell surfaces change differentially during looping [17] and that cell volumes enlarge differentially during ballooning [23], inspiring models in which changes in cell morphology drive the generation of cardiac left–right asymmetry [24] and chamber expansion [23]. It remains unclear which types of cellular changes underlie chamber curvature formation and how such changes are triggered.

Since cardiac contraction and blood flow begin at linear heart tube stages, chamber emergence and additional steps of cardiac morphogenesis take place while organ function is underway. Thus, biomechanical consequences of cardiac function, such as the fluid forces generated by circulating blood, have the potential to influence cardiac form (reviewed in [25]). Indeed, blockage of blood flow [26] or disruption of cardiac contractility [27] inhibits the formation of endocardial cushions at the boundary between the ventricle and the atrium, implicating biomechanical forces in the initiation of atrioventricular valve formation. Less is known about the impact of cardiac function on chamber curvature formation, although prior studies have indicated that chamber morphology is vulnerable to changes in functional parameters. For example, zebrafish and mouse mutants lacking atrial contractility also exhibit abnormal ventricular morphology, suggesting that blood flow through the ventricle somehow influences its shape and size [28,29]. However, the precise mechanisms by which functional inputs contribute to the control of chamber emergence are not yet understood.

Here, we address the open question of which cellular mechanisms underlie the formation of chamber curvatures and test their dependence upon functional influences. Taking advantage of the opportunities for high-resolution imaging of the developing zebrafish heart, we identify regionally confined cell shape changes as a key parameter in shaping chamber curvatures. Using zebrafish mutations that disrupt aspects of cardiac function, we determine that blood flow and contractility independently impact cell shape changes in the emerging ventricle. Thus, the acquisition of chamber shape via cell shape changes requires a balance between extrinsic and intrinsic biomechanical forces. Together, our data provide a model for the cellular mechanisms by which function can influence form during the definition of organ shape.

## Results

### *nppa* Expression Distinguishes Chamber Curvatures in Zebrafish

Chamber emergence occurs rapidly in the zebrafish embryo: the linear heart tube (LHT), which contracts and drives circulation by 24 h post-fertilization (hpf), transforms into two morphologically evident chambers, the ventricle and the atrium, by 48 hpf (Figure 1) [30]. As in other vertebrate hearts [10–12], each chamber is bean-shaped, with a distinct OC and IC (Figure 1A). In chick and mouse, expression of the *natriuretic peptide precursor a (nppa)* gene, also known as *atrial natriuretic factor (anf),* is restricted to the OC of the ventricle and atrium [10,31]. Similarly, we find that zebrafish *nppa* [28] marks OC myocardium as chambers emerge. Expression of *nppa* is first detectable by 24–28 hpf in a domain on the right side of the ventricular portion of the LHT (Figure 1C–1E, arrow) and a domain on the left side of the atrial portion of the LHT (Figure 1E, arrowhead). These domains are positioned to become the future ventricular and atrial OCs. As ballooning proceeds, *nppa* expression remains regionally restricted, being localized to the OC myocardium of both chambers while absent from the IC and atrioventricular canal regions (Figure 1G–1I). Thus, in parallel to the process observed in higher vertebrates, chamber emergence in zebrafish features the development of morphological and molecular differences between OC and IC myocardium.

### Regionally Confined Cell Shape Changes Accompany Chamber Emergence

**Figure 2:**
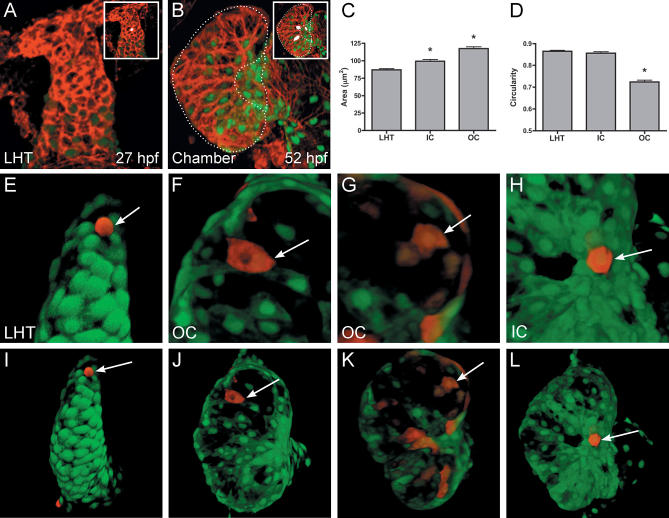
Regionally Confined Cell Shape Changes Accompany Chamber Emergence (A and B) Phalloidin staining (red) of wild-type hearts expressing *Tg(cmlc2:egfp)*. Insets show representative cell shapes filled in white. (C and D) Bar graphs depict surface area and circularity measurements of LHT, IC, and OC cells. Bar height indicates the mean value of a dataset, and error bars indicate standard error. An asterisk indicates statistically significant differences compared to LHT data (*p* < 0.0001). See Materials and Methods for details of morphometric analyses. (C) Surface area measurements in fixed samples demonstrate that IC and OC cells are significantly larger than LHT cells. (D) Cell shape assessments in fixed samples demonstrate that OC cells are significantly elongated relative to the more circular LHT and IC cells. (E–L) Confocal projections of live *Tg(cmlc2:egfp)-*expressing hearts that exhibit mosaic expression of *Tg(cmlc2:dsredt4)*. Arrows point to representative cells expressing both *dsredt4* and *egfp*. Three-dimensional assessment of cell morphologies in live embryos confirms that LHT (E and I) and IC (H and L) cells are relatively cuboidal, whereas OC cells are flattened and elongated (F, G, J, and K). OC cells are typically oriented with their long axes perpendicular to the arterial–venous axis (F and J), although some examples do not exhibit obvious orientation (G and K).

Despite the rapid emergence of the OC and IC during zebrafish heart development, it is not known which cellular mechanisms are responsible for establishing chamber curvatures during this time frame. We chose to test the hypothesis that the morphologically and molecularly distinct OC and IC regions also possess distinct cellular characteristics. Analyses of chick hearts have indicated that OC cells increase their apical surface area and volume during looping and ballooning [17,23]. To determine whether cell morphology changes contribute to the formation of chamber curvatures in zebrafish, we assessed the shape and size of cardiomyocytes as chambers emerge, focusing our analysis on the ventricle (Figure 2). We examined cell shape and surface area by using phalloidin to outline cells in embryos carrying the transgene *Tg(cmlc2:egfp),* which is expressed throughout the myocardium [32] (Figure 2A and 2B). Additionally, in order to view multiple cell surfaces and to examine cell morphology without fixation, we imaged live cells expressing *Tg(cmlc2:dsredt4)* mosaically within *Tg(cmlc2:egfp)*-expressing hearts (Figure 2E–2L; see Materials and Methods).

First, we examined cardiomyocyte morphology within the ventricular portion of the LHT (24–28 hpf; Figure 2A, 2C–2E, and 2I). Surface area measurements and shape assessments demonstrate that all cells in this region exhibit small and rounded morphologies (Figure 2A, 2C–2E, and 2I). Thus, even though *nppa* expression within the LHT anticipates formation of the OC (Figure 1D), cell morphologies are relatively uniform among the ventricular precursors at this stage. As development proceeds, ventricular cells increase their surface area (Figure 2C). Notably, however, only the cells in the OC of the expanded ventricle (48–58 hpf) significantly change their shape (Figure 2D). OC cells become flattened and elongated (Figure 2B, 2D, 2F, 2G, 2J, and 2K), and they are typically aligned with one another and oriented with their long axes perpendicular to the arterial–venous axis (Figure 2B, 2F, and 2J; Video S1), although occasionally they lack an obvious orientation (Figure 2B, 2G, and 2K; Video S2). Meanwhile, IC cells fail to flatten or elongate and instead maintain a cuboidal morphology (Figure 2B, 2D, 2H, and 2L; Video S3). We note that elongation is already apparent in some cells positioned to become OC at an intermediate stage of chamber emergence (36 hpf; unpublished data), suggesting that elongation of OC cells occurs gradually, concurrent with the gradual process of chamber expansion. We therefore propose that regionally confined cell shape changes underlie the acquisition of chamber morphology: the elongation and orientation of OC cells, coupled with the maintenance of cuboidal shape by IC cells, could account for the characteristic curvatures of the expanded ventricle.

### Blood Flow Promotes Ventricular Cell Enlargement and Elongation

**Figure 3:**
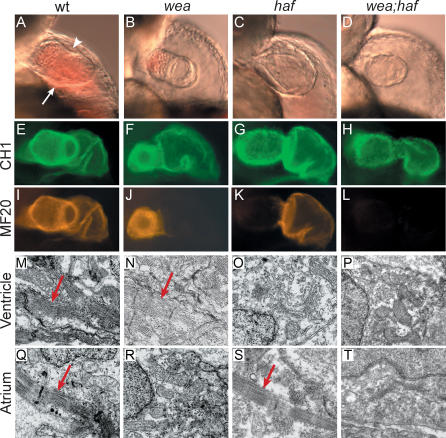
Mutation of *wea* and *haf* Cause Chamber-Specific Contractility Defects Analysis of *wea, haf,* and *wea;haf* double mutants at 48 hpf reveals abnormal ventricular morphology and chamber-specific sarcomere deficiencies. *haf^sk24^* and *wea^m58^* are recessive mutations that independently segregate; intercrosses of fish doubly heterozygous for *wea* and *haf* produce a 9:3:3:1 ratio of wild type:*wea*:*haf*:*wea;haf*. (A–D) Lateral views of live embryos, anterior to the top, ventricular plane of focus. (A) The wild-type (wt) ventricle possesses distinct concave (arrowhead) and convex (arrow) curvatures. (B) In contrast, the *wea* ventricle appears small, with less-pronounced curvatures. (C) The *haf* ventricle appears large and distended, with a notable separation between the myocardium and endocardium. (D) The *wea;haf* ventricle is small, with relatively spherical contours. (E–L) Whole-mount immunofluorescence detecting tropomyosin (CH1; green) and sarcomeric myosin heavy chain (MF20; red). Lateral views, ventricle to the left. Tropomyosin is present in all cardiomyocytes (E–H). In contrast, myosin heavy chain appears absent from the *wea* atrium (J), *haf* ventricle (K), and both chambers of the *wea;haf* heart (L). (M–T) Ultrastructural analysis of cardiomyocytes by transmission electron microscopy. Normal myofibril arrays (arrows) are present in wt chambers (M and Q), in the *wea* ventricle (N), and in the *haf* atrium (S). Organized myofibril arrays are absent from the *wea* atrium (R), the *haf* ventricle (O), and both chambers of the *wea;haf* heart (P and T).

**Figure 4:**
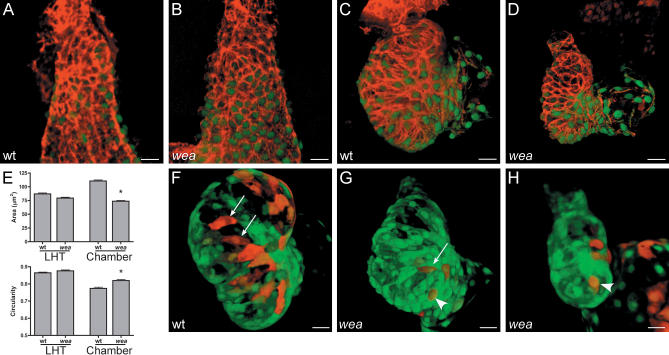
Cardiomyocytes in the *wea* Mutant Ventricle Retain Small Surface Areas and Fail to Elongate Normally (A–D) Phalloidin staining (red) of wild-type (wt) and *wea* mutant hearts expressing *Tg(cmlc2:egfp)* at LHT (A and B) and expanded chamber (C and D) stages. (E) Bar graphs depict surface area and circularity measurements, as in Figure 2. An asterisk indicates statistically significant differences compared to wt data (*p* < 0.0001). Since chamber curvatures are less distinct in *wea* mutants than they are in wt embryos, OC and IC data are pooled for comparison of cell size and shape at expanded chamber stages. Analysis of fixed samples demonstrates that *wea* cell surfaces are significantly smaller and more circular at chamber stages in comparison to wt. The trend toward smaller cell size in *wea* is also apparent at LHT stages (*p* < 0.05). (F–H) Confocal projections of live hearts, as in Figure 2, confirm the contrast between cell morphologies in wt and *wea* mutant ventricles. The wt OC typically contains elongated cells ([F] arrows), whereas *wea* OC cells have a smaller and less spread-out appearance, even when elongated ([G] arrow), and are frequently cuboidal ([G and H] arrowheads). Size bar represents 20 μm.

Because chamber emergence takes place following the onset of embryonic circulation, the physical forces inherent to cardiac function could influence the cell shape changes that accompany curvature formation. For example, normal blood flow, which creates changes in pressure and shear forces, has the potential to contribute to the induction of cellular enlargement and elongation at the OC. The zebrafish mutation *weak atrium (wea)* causes a phenotype appropriate for testing this hypothesis (Figure 3). The *wea* locus encodes *atrial myosin heavy chain (amhc,* also known as *myh6),* an atrium-specific myosin isoform [28]. Thus, *wea* mutants lack atrial sarcomeres, resulting in impaired atrial function and decreased blood flow through the ventricle compared with wild type ([28]; Figure 3M, 3N, 3Q, and 3R, and Videos S4 and S5). Although *amhc* is not expressed in the ventricle, ventricular shape and size are altered in *wea* mutants compared with wild type ([28], Figure 3A, 3B, 3E, 3F, 3I, and 3J). The *wea* mutant ventricle is small and dysmorphic, with less-pronounced curvatures than in the OC and IC of the wild-type ventricle. This secondary consequence of atrial dysfunction presumably indicates an impact of blood flow on ventricular morphology [28]. Because the cardiac cell number in the *wea* mutant ventricle is normal [28], the ventricle's abnormal morphology does not reflect a defect in cardiomyocyte proliferation. Instead, its aberrant curvatures could be the result of aberrant cell shapes or cellular organization. To investigate this possibility, we examined cardiomyocyte morphologies in the *wea* mutant ventricle (Figure 4).

**Figure 5:**
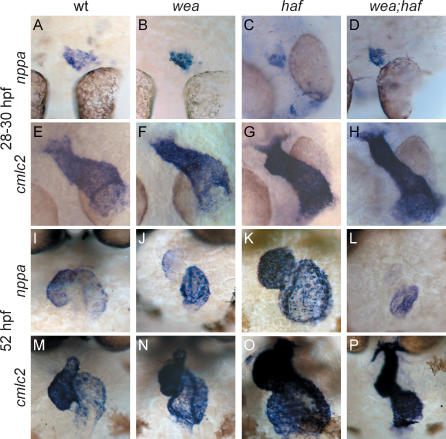
Regionalization of *nppa* Expression in *wea, haf,* and *wea;haf* Mutants Is Normal at LHT Stages but Abnormal at Expanded Chamber Stages Whole-mount in situ hybridization of *nppa* (A–D and I–L) compared to *cmlc2* (E–H and M–P). (A–H) Dorsal views, ventricle to the top, at LHT stages. (A, B, E, and F) are 28 hpf; (C, D, G, and H) are 30 hpf. Although initiation of *nppa* expression is delayed in *haf* (C) and *wea;haf* (D) mutants, the *nppa* expression domain is normal in the *wea* (B), *haf* (C), and *wea;haf* (D) LHT. (I–P) Frontal views, ventricle to the left, at expanded chamber stages (52 hpf). (J) Regionalized *nppa* expression is not maintained in the *wea* mutant ventricle; instead, *nppa* expression in the *wea* ventricle is fainter and in a smaller domain than in wild type (wt) (I). (K) In the *haf* mutant ventricle, intense *nppa* expression is found throughout the chamber, rather than being restricted to the OC. (L) The *wea;haf* double mutant ventricle exhibits weak and diffuse *nppa* expression.

In the *wea* LHT, cell shapes are uniform, round, and indistinguishable from those of wild type, although measurements of surface area suggest a trend toward smaller cell size (Figure 4A, 4B, and 4E). By the time of chamber emergence, the surface area of individual cells throughout the ventricle is significantly smaller than in wild type (Figure 4C–4E). Although elongated cells can be found in the *wea* OC, those cells tend to be less elongated than cells in the wild-type OC (Figure 4C–4H). In addition, cuboidal cell morphologies, normally confined to the IC, are frequently found in the *wea* OC (Figure 4G and 4H). The persistence of small, cuboidal cells in the *wea* ventricle does not reflect a simple developmental delay, because we detect similar cell morphologies in *wea* mutants at 72 hpf (H. J. Auman and D. Yelon, unpublished data). Additionally, treatment with 5 mM 2,3-butanedione monoxime (BDM), another method for reducing blood flow in zebrafish embryos [27], has an effect on ventricular cardiomyocyte morphology similar to that observed in *wea* mutants: at 48–52 hpf, BDM-treated ventricular cells are smaller (83 ± 2 μm^2^ surface area) and less elongated (0.82 ± 0.01 circularity) than wild-type ventricular cells (Figure 4E; 110 ± 2 μm^2^ surface area, 0.77 ± 0.01 circularity; both significantly different from BDM-treated values, *p* < 0.0001). The reduced degree of elongation observed in the *wea* OC correlates with a reduction in *nppa* expression at this location (Figure 5). Regionalized *nppa* expression initiates normally in the *wea* LHT (Figure 5A, 5B, 5E, and 5F), but, by 52 hpf, ventricular *nppa* expression is weak and occupies a smaller proportion of the chamber in comparison to wild-type (Figure 5I, 5J, 5M, and 5N). Together, these data suggest that normal blood flow is needed to promote the cell enlargement, cell elongation, and regionalized gene expression that accompany chamber curvature formation.

### Contractility Restricts Ventricular Cell Size and Elongation

**Figure 6:**
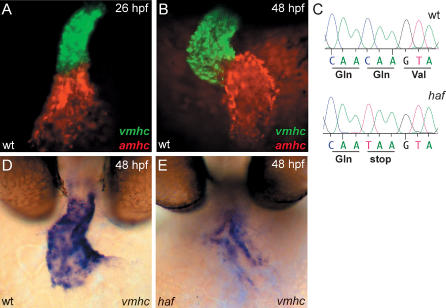
*haf^sk24^* Is a Strong Loss of Function Allele of *vmhc,* a Ventricle-Specific Myosin Heavy Chain Gene with Expression Complementary to *amhc* (A and B) Fluorescent in situ hybridization for *vmhc* (green) and *amhc* (red) in wild-type (wt) embryos at LHT ([A] 26 hpf, dorsal view) and expanded chamber ([B] 48 hpf, frontal view) stages. At all stages examined, *vmhc* is expressed in the ventricular myocardium [51]. In contrast, *amhc* expression is restricted to the atrial myocardium [28]. (C) Comparison of *vmhc* coding sequence in wt and *haf^sk24^* embryos reveals a C to T transition at position 3,094 that results in a premature stop codon in *haf^sk24^*. (D and E) Whole-mount in situ hybridization depicts frontal view of *vmhc* expression in wt and *haf* at 48 hpf. *vmhc* expression is dramatically reduced in the *haf* mutant ventricle.

As blood flow promotes cell enlargement, we hypothesized that an opposing mechanism might restrict or moderate cell size. We reasoned that a good candidate for such resistance would be a feature intrinsic to the myocardial cell, such as its contractility. To test this idea, we utilized a mutation in the zebrafish *ventricular myosin heavy chain* gene *(vmhc,* also known as *myh7)* that blocks contractility in ventricular cardiomyocytes (Figure 6). The ventricle-specific expression of *vmhc* complements the atrium-specific expression of *amhc* ([28,33] and Figure 6A and 6B). The *half-hearted (haf)* mutation (see Materials and Methods) creates a premature stop codon in *vmhc* that is predicted to result in a truncated and non-functional Vmhc protein (Figure 6C). As a result, *haf* mutants lack ventricular contractility and provide a chamber-specific functional complement to *wea* mutants (Figure 3O and 3S; Video S6).

**Figure 7:**
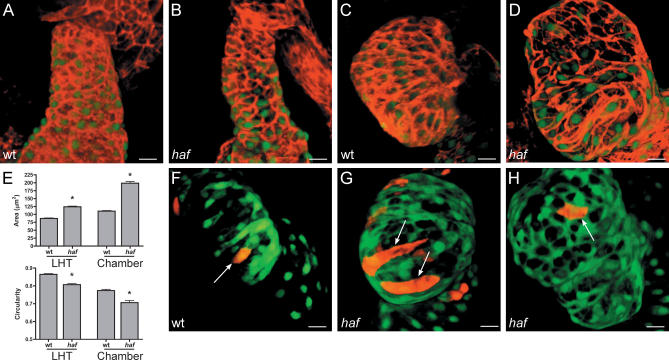
Cardiomyocyte Surfaces throughout the *haf* Ventricle Become Excessively Enlarged and Elongated (A–D) Phalloidin staining (red) of wt and *haf* mutant hearts expressing *Tg(cmlc2:egfp)* at LHT (A and B) and expanded chamber (C and D) stages. (E) Bar graphs depict surface area and circularity measurements, as in Figure 2. An asterisk indicates statistically significant differences compared to wild-type (wt) data (*p* < 0.0001). The shape and size of *haf* cells are significantly different from those of wt cells; at both LHT and expanded chamber stages, *haf* cells are larger and more elongated. (F–H) Confocal projections of live hearts, as in Figure 2, confirm the abnormal size and shape of *haf* mutant cardiomyocytes in the expanded ventricle. *haf* cells ([G and H] arrows) are larger than their wt counterparts ([F] arrow) and can be greatly elongated (G). Size bar represents 20 μm.

In contrast to the small ventricle of *wea* mutants, the *haf* mutant ventricle is large and distended, with abnormally spherical contours (Figure 3C). As for *wea* mutants, we found that cardiac cell number in *haf* mutants is normal (at 48 hpf, 231 ± 3 cells in wild type vs. 229 ± 13 in *haf, n* = 4), suggesting that aberrant cell shapes, rather than aberrant proliferation, might underlie the development of abnormal ventricular curvatures in *haf*. Indeed, examination of cell morphology in the *haf* ventricle revealed a significant increase in cell surface area, which is already apparent in the LHT and becomes more pronounced during chamber emergence (Figure 7A–7E). Many cells within the expanded *haf* ventricle are also highly elongated (Figure 7C–7G), although not all are elongated to the same extent (Figure 7H). As in wild type, the orientation of elongated cells in *haf* is typically perpendicular to the arterial–venous axis. However, rather than being restricted to the OC, elongated cells are often found throughout the chamber. The overextension and overrepresentation of elongated cells in the *haf* ventricle correlate with the increased and widespread expression of *nppa* throughout the chamber (Figure 5K). In contrast, *nppa* expression is correctly regionalized in the *haf* LHT, despite a slight delay in initiation of expression (Figure 5C). Overall, these data indicate that Vmhc function is required to restrict cell size, to limit the extent of cellular elongation, and to confine the distribution of elongated cell types during chamber curvature formation.

### Intrinsic Contractility Independently Regulates Cardiomyocyte Morphology

**Figure 8:**
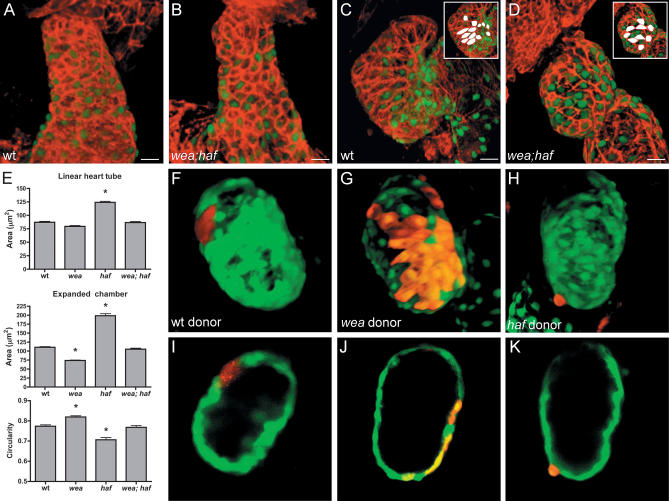
Cells Lacking Vmhc Assume Different Morphologies under Different Hemodynamic Conditions (A–D) Phalloidin staining (red) of wild-type (wt) and *wea;haf* double mutant hearts expressing *Tg(cmlc2:egfp)* at LHT (A and B) and expanded chamber (C and D) stages. Insets highlight representative cell shapes in white. *wea;haf* cells are irregularly shaped and lack apparent organization or alignment. (E) Bar graphs depict surface area and circularity measurements, as in Figure 2. An asterisk indicates statistically significant differences compared to wt data (*p* < 0.0001). Despite their irregular organization and morphology, *wea;haf* ventricular cells exhibit a size and circularity range similar to that of wt cells and distinct from that of *wea* or *haf*. Notably, *wea;haf* ventricular cells enlarge more than *wea* cells do (*p* < 0.0001), even though *wea;haf* mutants lack blood flow. Also, *wea;haf* cell morphologies are not as extreme in size (*p* < 0.0001) or elongation (*p* < 0.001) as *haf* cell morphologies, even though *wea;haf* mutant cells lack Vmhc. (F–K) Chimeric ventricles resulting from transplantation of rhodamine dextran-labeled blastomeres into wt hosts expressing *Tg(cmlc2:egfp)*. Optical sections of (F, G, and H) are shown in (I, J, and K), respectively. (F, G, I, and J) Cells from wt or *wea* donors integrate normally within the wall of the wt host ventricle. (H and K) In contrast, cells from *haf* donors project abnormally from the ventricular wall and fail to maintain normal shape, indicating a cell-autonomous requirement for Vmhc in the maintenance of cardiomyocyte morphology. Size bar represents 20 μm.

Our analysis of the *haf* mutant phenotype indicates that ventricular cell shapes are significantly affected by the loss of Vmhc function, raising the possibility that cardiomyocyte contractility directly influences cardiomyocyte morphology. However, it is also possible that the particular hemodynamic environment in *haf* mutants could have an indirect effect on ventricular cell shape. Although the *haf* ventricle is noncontractile, the *haf* atrium contracts normally (Video S6). As a result, blood fills the *haf* ventricle without subsequent ventricular response. Thus, *haf* ventricular cells are likely subjected to an elevation in preload that could potentially lead to secondary effects on cell shape. We therefore wished to uncouple the influences of blood flow and contractility in the establishment of cell shapes, and proceeded to test whether the morphology of a noncontractile ventricular cell changes when placed in different hemodynamic conditions (Figure 8).

The *wea;haf* double mutant, which lacks both atrial and ventricular contractility (Figure 3L, 3P, and 3T; Video S7), enabled us to test the role of *vmhc* in the acquisition of ventricular cell shape without the complicating factor of atrial contraction and elevated preload. Although the *wea;haf* ventricle is typically not as distended as the *haf* ventricle, both ventricles have a relatively spherical appearance (Figure 3C and 3D). Cell shape and size are normal in the *wea;haf* LHT; the double mutants exhibit neither the large cells of the *haf* LHT nor the trend toward small cells seen in the *wea* LHT (Figure 8A, 8B, and 8E). However, cells in the *wea;haf* expanded ventricle are clearly abnormal: they are irregularly shaped and lack apparent alignment, consistent orientation, or regional organization (Figure 8C and 8D). The lack of a particular area of cellular elongation in the *wea;haf* ventricle correlates with its diffuse, weak *nppa* expression (Figure 5I and 5L). Strikingly, unlike *wea* cells, which maintain small size and cuboidal morphology within the expanded chamber, *wea;haf* cells do enlarge and elongate, despite an absence of blood flow. Indeed, although they exhibit irregular morphologies, *wea;haf* cells exhibit an average surface area and circularity similar to that of wild-type cells (Figure 8E). Therefore, *wea;haf* ventricular cells seem to lack an intrinsic mechanism for maintenance of cell morphology.

To test this idea further, we challenged the shape of *haf* cells by placing them into a hemodynamic environment in which they would be subjected to normal flow, as is found within the wild-type ventricle. We transplanted *haf* mutant cells into wild-type hosts at midblastula stages and analyzed the morphology of resultant *haf* ventricular clones in the expanded chamber. In this context, *haf* cells adopt an entirely different and unusual morphology: whether in the OC or IC, the noncontractile *haf* donor cells round up and extrude from the contractile ventricular wall (Figure 8H and 8K). This protrusion phenotype, seen in four out of four embryos, was never observed when wild-type (*n* > 10 embryos) or *wea* (*n* > 12 embryos) cells were transplanted in control experiments (Figure 8F, 8G, 8I, and 8J). These results demonstrate that Vmhc is required cell-autonomously for maintenance of cardiomyocyte morphology and chamber wall integrity. Additionally, our *haf, wea;haf,* and transplantation data argue that the particular irregular shape adopted by a cell lacking Vmhc depends upon the contractility of its neighbors and the nature of its hemodynamic environment. Together, our data indicate that maintenance of appropriate cardiomyocyte size and shape requires cell-autonomous traits that are dependent on contractility and independent of the influence of blood flow.

## Discussion

### Cell Shape Changes Underlying Chamber Emergence Require a Balance of Biomechanical Forces

Together, our analyses provide a new model for the cellular processes creating chamber shape. During ventricular chamber emergence, regionally confined cell shape changes correlate with the locations of curvature formation. We therefore propose that cardiomyocyte elongation plays a substantial role in creating the characteristic convex shape of the OC; on the opposing side of the chamber, maintenance of cuboidal cardiomyocyte morphology contributes to creating the concave IC. Blood flow through the developing ventricle promotes cardiomyocyte elongation and thereby encourages establishment of the OC. The limits of cardiomyocyte elongation are constrained by the cell's intrinsic contractility, and these constraints are critical for maintaining the IC and avoiding chamber dilation. Thus, a balance between extrinsic and intrinsic functional inputs is important for the control of cardiomyocyte shape and, consequently, chamber shape.

The proposed balance between the functional inputs of flow and contractility is likely to reflect a balance between opposing biomechanical forces. Although our experiments do not address the specific physical forces impacting chamber curvature, our data are consistent with a model in which equilibrium between fluid forces and cellular tension influences cardiomyocyte morphology. Fluid forces could affect cardiomyocyte shape in several ways. Sensation of shear forces by the endocardium could indirectly trigger cellular deformation in the myocardium. Endothelial cells are known to respond to shear forces via changes in gene expression in vitro [34,35] and in vivo [36] and, in zebrafish, shear forces are predicted to exist at levels detectable by endocardium at 37 hpf, when chamber emergence is underway [26]. Alternatively, hemodynamic pressure could play a more direct role in stretching cardiomyocytes. We favor this idea, since the flattened, elongated, and aligned morphologies of OC cells are reminiscent of the shapes assumed by stretched cardiomyocytes in culture [37,38].

Although fluid forces seem to encourage distortion of cardiomyocyte dimensions, cardiomyocyte contractility provides resistance to cell shape change, perhaps by producing inherent cellular tension. This tension could result from the elastic nature of actively contracting sarcomeres. Alternatively, sarcomere assembly could contribute to the organization of actomyosin cytoskeletal structures that create cellular stiffness. In either case, our data indicate that sarcomere integrity is critical for the maintenance of cardiomyocyte morphology. This conclusion is compatible with a previous study in zebrafish demonstrating that transplanted cells lacking the giant protein Titin have difficulty integrating into the wild-type chamber wall [39]. Titin is a multidomain protein with several proposed functions [40]; our results suggest that the protrusion phenotype observed in cells lacking Titin is related to the role of Titin in sarcomere organization.

Although both IC and OC cells respond to functional inputs, it is clear that they respond differently, such that elongation occurs only in the region of the OC. We propose that intrinsic patterning programs evident in the LHT create differences among future IC and OC cells, perhaps conferring differential vulnerability to functional inputs. These initial differences could be the predecessors of the distinct mechanical properties of each curvature, such as the greater stiffness of the IC observed in the chick heart [41]. Our examination of *nppa* expression indicates a molecular distinction between the future IC and OC cells within the LHT, before cell shape changes begin. This initial regionalization of *nppa* expression, apparent in the LHT of *wea* mutants, *haf* mutants, and *wea;haf* mutants, is evidently not dependent on either blood flow or contractility (Figure 5A–5D). In contrast, later *nppa* expression in the expanded chamber appears to relate to functional inputs, responding as cells do to the forces promoting elongation. As stretch is a primary inducer of *nppa* expression [42,43], expression of *nppa* in the OC may correlate with increased levels of stretch in those cells. Together, our data suggest a model in which organ shape is the product of cell shape derived from the integration of patterning programs with balanced physical forces. The interplay between extrinsic forces and intrinsic cellular properties has the potential to influence organ shape acquisition even in noncontractile tissues, particularly those that experience biomechanical inputs generated by circulating fluids (e.g., kidney tubules [44] or brain ventricles [45]).

### Implications for the Origins of Cardiomyopathy

Our proposed model is likely to be relevant to the mechanisms underlying cardiomyopathy. Mutation of the human cardiac myosin heavy chain genes *MYH6* or *MYH7* can cause hypertrophic cardiomyopathy [46,47], and mutation of *MYH7* can cause dilated cardiomyopathy [38]. It is not yet understood how the various mutations identified lead to the diversity of phenotypes observed in patients [48]. Here, we demonstrate two distinct cellular mechanisms by which mutation of myosin heavy chain genes can create abnormal ventricular morphology: a cell-autonomous mechanism via mutation of *vmhc* and a cell non-autonomous mechanism via mutation of *amhc*. Furthermore, our model suggests that even subtle modification of circulation or contractility could lead to abnormalities in cell morphology with significant consequences for chamber morphology. Thus, our findings broaden the array of mechanisms to consider as cellular etiologies of cardiomyopathy.

## Materials and Methods

### Immunofluorescence and F-actin staining.

Whole-mount immunostaining was performed as previously described [49], using primary antibodies against sarcomeric myosin heavy chain (MF20) and tropomyosin (CH1). MF20 and CH1 were obtained from the Developmental Studies Hybridoma Bank (Iowa City, Iowa, United States), maintained by the Department of Biological Sciences, University of Iowa, under contract NO1-HD-2–3144 from the National Institute of Child Health and Human Development (NICHD). Secondary antibodies were obtained from Southern Biotech (Birmingham, Alabama, United States). We visualized cell outlines with rhodamine-conjugated phalloidin (Molecular Probes, Eugene, Oregon, United States) at a dilution of 1:75, using a protocol that preserves GFP fluorescence in fixed embryos [50].

### In situ hybridization.

Probes for *cmlc2, vmhc, amhc,* and *nppa* [28,51] were used as previously described for standard in situ hybridization. For fluorescent in situ hybridization, probes were labeled with digoxygenin (Roche, Basel, Switzerland), fluorescein (Roche), or dinitrophenol (Mirus, Madison, Wisconsin, United States) and were detected by deposition of fluorescein or Cy3 tyramides (PerkinElmer, Wellesley, Massachusetts, United States). A detailed protocol for fluorescent in situ hybridization will be published separately (J. Schoenebeck, B. Keegan, and D. Yelon, unpublished data).

### Mosaic labeling of cardiomyocytes.

Embryos homozygous for *Tg(cmlc2:egfp)* were injected at the one-cell stage with 5 pg of *Tg(cmlc2:dsredt4)* plasmid DNA. The *Tg(cmlc2:dsredt4)* transgene drives expression of *dsredt4* [52] with an approximately 0.8-kb fragment found upstream of the 5′ UTR of *cmlc2* (−870/+3; [32]). Injected embryos typically had zero to ten cardiomyocytes expressing both *dsredt4* and *egfp*. Embryos of interest were mounted in viewing chambers with 0.2% tricaine to anesthetize the embryo and minimize cardiac contraction during confocal imaging.

### Imaging.

Embryos were examined with Zeiss M2Bio and Axioplan microscopes and photographed with a Zeiss Axiocam digital camera (Carl Zeiss, Oberkochen, Germany). Images were processed using Zeiss Axiovision and Adobe Photoshop software (Adobe Systems, San Jose, California, United States). Live embryo videos were recorded and processed using an Optronics DEI750 video camera, a Zeiss M2Bio microscope, and iMovie software. Transmission electron microscopy procedures were conducted as previously described [28]. Confocal images were obtained using a Zeiss LSM510 confocal laser-scanning microscope and LSM software. Z-stacks were rendered in 3D and analyzed with Volocity software (Improvision, Coventry, United Kingdom).

### Morphometrics.

We used ImageJ software (National Institutes of Health [NIH] http://rsb.info.nih.gov/ij/) to trace cell outlines and measure each cell's surface area and perimeter. Cells were chosen for measurement when their outlines were clearly visible and sharply rendered in the *xy* plane of view. Circularity was calculated by ImageJ as a normalized ratio of area (A) to perimeter (P), with a ratio of 1 representing a perfect circle (circularity = 4πA/P^2^). In this way, the circularity value distinguishes cells with more circular surfaces from those with more elliptical or elongated surfaces.

Measurements of cells from multiple embryos at the LHT stage (24–28 hpf) and at the expanded chamber stage (48–58 hpf) were pooled into datasets. When analyzing the LHT, we measured cells throughout the outflow half of the tube, thereby including cells that will contribute to the future ventricular IC and OC. In the expanded wild-type ventricle (48–58 hpf), the IC and OC could easily be distinguished morphologically. Because the curvatures were distorted in mutant or BDM-treated embryos, we compared cardiomyocytes throughout the mutant or BDM-treated ventricle to pooled data from wild-type ventricular cardiomyocytes. Sample sizes were as follows: wild-type LHT, 173 cells from 11 embryos; wild-type chamber, 336 cells from 18 embryos; wild-type IC, 122 cells from 14 embryos; wild-type OC, 210 cells from 16 embryos; *wea* LHT, 99 cells from four embryos; *wea* chamber, 250 cells from 17 embryos; BDM-treated chamber, 99 cells from 14 embryos; *haf* LHT, 215 cells from 13 embryos; *haf* chamber, 149 cells from seven embryos; *wea;haf* LHT, 114 cells from six embryos; and *wea;haf* chamber, 161 cells from ten embryos. Area and circularity values were compared using unpaired *t*-tests.

### Identification of the *half-hearted* mutation.

We isolated the *half-hearted (haf^sk24^)* mutation in our laboratory's ongoing screen for ethylnitrosourea-induced mutations that disrupt chamber form and function (F. Olale, T. Bruno, A. Schier, and D. Yelon, unpublished data). In this screen, we analyzed the haploid progeny of F1 females at 30–48 hpf using established protocols for live imaging and immunofluorescent detection of sarcomeric myosin heavy chain [49]. *haf* mutants were evident during screening because of their noncontractile and dysmorphic ventricles. Corresponding with the lack of ventricular contractility, immunolocalization of sarcomeric myosin heavy chain was absent from the *haf* mutant ventricle (Figure 3K), and ultrastructural analyses via electron microscopy revealed an absence of ventricular sarcomeres (Figure 3O). Linkage analysis demonstrated that the *haf* mutation is located near the SSLP marker Z13475 on Chromosome 2 (one recombinant in 50 meioses), near the *vmhc* gene [28]. Sequencing of genomic DNA in *haf* mutants identified a C to T transition at position 3,094 of the *vmhc* open reading frame, creating a premature stop codon (Figure 6C). The wild-type Vmhc protein contains 1,937 amino acids, and the predicted *haf* Vmhc protein would terminate after amino acid 1,058, disrupting the myosin tail domain to result in a nonfunctional truncated product. Furthermore, in situ hybridization demonstrated a marked decrease of *vmhc* transcript in the *haf* mutant ventricle (Figure 6D and 6E), suggesting degradation of the mutant transcript by nonsense-mediated decay. Thus, protein production is unlikely, consistent with the loss of detectable ventricular myosin heavy chain (Figure 3K). Taken together, these analyses indicate that the *haf* locus encodes Vmhc.

### Transplantation.

We used standard transplantation techniques at midblastula stages [53]. To create each chimeric embryo, we placed 10–15 blastomeres from a donor embryo near the embryonic margin of a host embryo. For transplant hosts, we used wild-type embryos homozygous for *Tg(cmlc2:egfp)*. Transplant donors were generated from intercrosses of *wea* or *haf* heterozygotes carrying *Tg(cmlc2:egfp)* and injected with rhodamine dextran (Molecular Probes) as a lineage tracer. Donor embryos were retained for retrospective identification as mutant or wild-type based on phenotypic analysis at 48 hpf. We observed no differences in viability among transplanted cells for all genotypes.

## Supporting Information

Video S1 Example of an Elongated OC Cell within the Ventricle Video shows an example of mosaically labeled *egfp*- and *dsredt4*-expressing cardiomyocytes rendered in three dimensions and rotated for alternate views. Video corresponds to the cell highlighted in Figure 2F and 2J. Arrow in frame 1 points to the cell of interest. Scale is indicated in the lower right corner.
(1.6 MB MOV)

Video S2 Example of a Flattened, Non-Oriented OC Cell Video corresponds to the cell highlighted in Figure 2G and 2K. Format as described for Video S1.
(7.2 MB MOV)

Video S3 Example of a Cuboidal Cell of the IC Video corresponds to the cell highlighted in Figure 2H and 2L. Format as described for Video S1.
(6.9 MB MOV)

Video S4 Wild-Type Embryo at 48 hpf Demonstrating Normal Cardiac Chamber Contractions Video of live wild-type 48-hpf embryo carrying *Tg(cmlc2:egfp);* both chambers contract. Ventricle is to the left.
(5.2 MB MOV)

Video S5 Atrial Contractile Deficit in *wea*
Video of live *wea* 48-hpf embryo carrying *Tg(cmlc2:egfp);* the ventricle contracts and the atrium beats weakly. Ventricle is to the left.
(6.0 MB MOV)

Video S6 Ventricular Contractile Defect in *haf*
Video of live *haf* 48-hpf embryo carrying *Tg(cmlc2:egfp);* the ventricle is noncontractile and the atrium contracts. Ventricle is to the left.
(7.0 MB MOV)

Video S7 The *wea;haf* Heart Is Noncontractile Video of live *wea;haf* 48-hpf embryo carrying *Tg(cmlc2:egfp);* neither chamber contracts. Ventricle is to the left.
(3.7 MB MOV)

### Accession Numbers

The GenBank (http://www.ncbi.nlm.nih.gov/Genbank) accession numbers for the genes discussed in this paper are *myh6* (NM_198823) and *vmhc* (AF114427).

## Abbreviations

hpf: hours post-fertilization
IC: inner curvature of the cardiac chamber
LHT: linear heart tube
OC: outer curvature of the cardiac chamber
